# Prevalence, risk factors for infection and subtype distribution of the intestinal parasite *Blastocystis* sp. from a large-scale multi-center study in France

**DOI:** 10.1186/s12879-016-1776-8

**Published:** 2016-08-26

**Authors:** Dima El Safadi, Amandine Cian, Céline Nourrisson, Bruno Pereira, Christelle Morelle, Patrick Bastien, Anne-Pauline Bellanger, Françoise Botterel, Ermanno Candolfi, Guillaume Desoubeaux, Laurence Lachaud, Florent Morio, Christelle Pomares, Meja Rabodonirina, Ivan Wawrzyniak, Frédéric Delbac, Nausicaa Gantois, Gabriela Certad, Laurence Delhaes, Philippe Poirier, Eric Viscogliosi

**Affiliations:** 1 Université de Lille, CNRS, Inserm, CHU Lille, Institut Pasteur de Lille, U1019 – UMR 8204 – CIIL – Centre d’Infection et d’Immunité de Lille, 1 rue du Professeur Calmette, BP 245, 59019 Lille cedex, France; 2 Laboratoire de Parasitologie-Mycologie, CHU Gabriel-Montpied, Clermont-Ferrand, France; 3 Clermont Université, Université Blaise Pascal-Université d’Auvergne - CNRS UMR 6023 Laboratoire Microorganismes: Génome et Environnement, Clermont-Ferrand, France; 4 CHU Clermont-Ferrand, Unité de Biostatistiques, Direction de la Recherche Clinique (DRCI), Clermont-Ferrand, France; 5 Laboratoire de Parasitologie-Mycologie, CHU de Montpellier, CNRS UMR 5290/IRD 224/UM1, Université de Montpellier 1, Montpellier, France; 6 Laboratoire de Parasitologie-Mycologie, CHU de Besançon, Besançon, France; 7 Laboratoire de Parasitologie-Mycologie, AP-HP Hôpital Henri Mondor, Créteil, France; 8 Institut de Parasitologie et de Pathologie Tropicale de Strasbourg, Université de Strasbourg, Hôpitaux Universitaires de Strasbourg, Strasbourg, France; 9 Service de Parasitologie-Mycologie-Médecine Tropicale, CHU de Tours/CEPR Inserm U1100 Equipe 3, Université François-Rabelais de Tours, Tours, France; 10 Département de Parasitologie-Mycologie, Faculté de Médecine de Montpellier-Nîmes, Université de Montpellier I, CHU de Montpellier, Montpellier, France; 11 Département de Parasitologie et Mycologie Médicale, Laboratoire de Parasitologie-Mycologie, Institut de Biologie, CHU de Nantes, EA1155-IICiMed, Université de Nantes, Nantes, France; 12 Laboratoire de Parasitologie-Mycologie CHU de Nice, C3M INSERM U1065, Université de Nice Sophia Antipolis, Nice, France; 13 Service de Parasitologie, Hospices Civils de Lyon, Lyon, France; 14 Département de Parasitologie-Mycologie, CHU de Lille, Faculté de Médecine, Lille, France

**Keywords:** *Blastocystis* sp, Intestinal parasite, Molecular epidemiology, PCR, Subtyping, Risk factors for infection

## Abstract

**Background:**

*Blastocystis* sp. is the most common intestinal parasite of humans. Despite its potential public health impact, epidemiological data regarding the prevalence and molecular subtype distribution of *Blastocystis* sp. in Europe are rarely reported. Therefore, the first multi-center epidemiological survey performed in Europe was conducted in France to diagnose and subtype *Blastocystis* sp. and to identify risk factors for infection.

**Methods:**

Stool samples from 788 patients were collected either in summer or winter in 11 hospitals throughout France together with patient data. All stool samples were tested for the presence of *Blastocystis* sp. by quantitative PCR targeting the SSU rDNA gene. Positive samples were sequenced to determine the distribution of the subtypes in our cohort. Statistical analyses were performed to identify potential risk factors for infection.

**Results:**

Using quantitative PCR, the overall prevalence of *Blastocystis* sp. was shown to reach 18.1 %. The prevalence was significantly higher in summer (23.2 %) than in winter (13.7 %). Travellers or subjects infected with other enteric parasites were significantly more infected by *Blastocystis* sp. than non-travellers or subjects free of other enteric parasites, respectively. Different age-related epidemiological patterns were also highlighted from our data. The prevalence of *Blastocystis* sp. was not significantly higher in patients with digestive symptoms or diagnosed with chronic bowel diseases. Among symptomatic patients, *Blastocystis* sp. infection was significantly associated with abdominal pain. Gender, socioeconomic status, and immune status were not identified as potential risk factors associated with infection. Among a total of 141 subtyped isolates, subtype 3 was predominant (43.3 %), followed by subtype 1 and subtype 4 (20 %), subtype 2 (12.8 %), subtype 6 and subtype 7 (2.1 %). No association between ST and clinical symptoms was statistically evidenced.

**Conclusions:**

A high prevalence of *Blastocystis* sp. infection was found in our French patient population. Seasonal impact on the prevalence of *Blastocystis* sp. was highlighted and recent travels and age were identified as main risk factors for infection. Most cases were caused by subtypes 1 to 4, with a predominance of subtype 3. Large variations in both prevalence and ST distribution between hospitals were also observed, suggesting distinct reservoirs and transmission sources of the parasite.

**Electronic supplementary material:**

The online version of this article (doi:10.1186/s12879-016-1776-8) contains supplementary material, which is available to authorized users.

## Background

*Blastocystis* sp. is a common protozoan intestinal parasite with worldwide distribution that inhabits the digestive tract of humans and a large variety of animal hosts [1–3]. In numerous epidemiological surveys, this cosmopolitan enteric parasite was frequently identified as the most common unicellular eukaryote found in human fecal samples [1, 2, 4]. Indeed, its prevalence may reach 20 % in industrialized countries, including the European population [5] and 50 % in developing countries [6]. Recently, the prevalence of *Blastocystis* sp. was shown to be 100 % in a cohort of children living in a rural area in Senegal, highlighting the impact of blastocystosis mainly in developing countries with poor healthcare and hygiene [7]. In this regard, a higher prevalence of this parasite was found among European people with a history of recent travel to tropical countries [5]. At the morphological level, four major forms of *Blastocystis* sp. have been described, including the infective cyst which is able to survive for a long period in feces and environmental sources and is resilient to standard water chlorination, facilitating waterborne transmission of the parasite [1, 8]. Therefore, the fecal-oral route is considered the main mode of transmission of *Blastocystis* sp. through the consumption of food or water contaminated by cysts.

Blastocystosis is usually diagnosed using direct-light microscopy of fecal smears or possibly short-term xenic in vitro culture of stool samples. However, these methods have a low diagnostic sensitivity compared with molecular tools, i.e. PCR assays, and could greatly underestimate the real prevalence of the parasite [9]. A remarkable genetic diversity has been revealed among *Blastocystis* sp. isolates from humans and other animals based on the comparison of small subunit (SSU) rRNA gene sequences. Consequently, seventeen lineages of so-called subtypes (ST1 to ST17) (arguably separate species) have been identified among mammalian and avian isolates [10], nine of which (ST1 to ST9) are found in humans with varying prevalence [2, 4]. The other STs (ST10-ST17) are exclusively found in animals [10]. Based on a recent review including all human samples subtyped thus far across various geographic regions worldwide [4], approximately 90 % of human isolates belonged to ST1 to ST4, with a predominance of ST3 (around 60 % of these isolates). Even though these four STs were found in different animal hosts, their predominance in the human population is likely explained by large-scale human-to-human transmission [1, 2]. To our knowledge, ST9 was restricted to humans and until now has been identified in only 3 people from Denmark and Japan [4]. ST5 to ST8 supposedly of animal origin were rarely found in humans and their presence might be linked to zoonotic transmission. Besides, a higher risk of *Blastocystis* sp. infection was found in people with close animal contact, including zoo keepers [11].

The human health impact of *Blastocystis* sp. still remains uncertain because the parasite is frequently found in asymptomatic patients and has been associated with a wide range of non-specific symptoms including diarrhea, abdominal pain, bloating, nausea, and vomiting as well as urticarial lesions [1–3]. However, recent findings using in vitro and in vivo approaches combined with *in silico* analysis of genomic data and clinical reports strongly suggested the pathogenic potential of *Blastocystis* sp. by identifying putative virulence factors such as cysteine proteases. These proteases are secreted by the parasite and can induce epithelial barrier dysfunction [1–3, 12–14]. The proposed models for pathogenesis of *Blastocystis* sp. [13–16] mainly involved adhesion of parasites to the intestinal epithelium, apoptosis and degradation of tight junction proteins of intestinal epithelial cells resulting in increased intestinal permeability, degradation of IgA and induction of a pro-inflammatory cytokine response. *Blastocystis* sp. was also recently associated with Irritable Bowel Syndrome (IBS) [16, 17], a multifactorial functional bowel disorder partly explained by dysbiosis [18].

All these new data provide evidence that the public health burden of *Blastocystis* sp. continues to be underestimated, hence the interest in conducting large-scale epidemiological surveys in industrialized countries. In France, very little data were available concerning both the prevalence and ST distribution of *Blastocystis* sp. The parasite was previously reported in two French cohorts with a respective prevalence of 3.0 and 6.1 % by direct-light microscopy of fecal smears [19, 20]. In addition, conflicting ST distributions were observed between two French molecular studies conducted in different geographic areas and both including a limited number of samples [9, 21]. Therefore, the aim of the present study was to reinforce the picture of *Blastocystis* sp. prevalence and molecular ST distribution in Europe by performing the first multi-center survey conducted in France from a large cohort of patients carried out between December 2012 and September 2013.

## Methods

### Cohort of patients and collection of samples

**Fig. 1:**
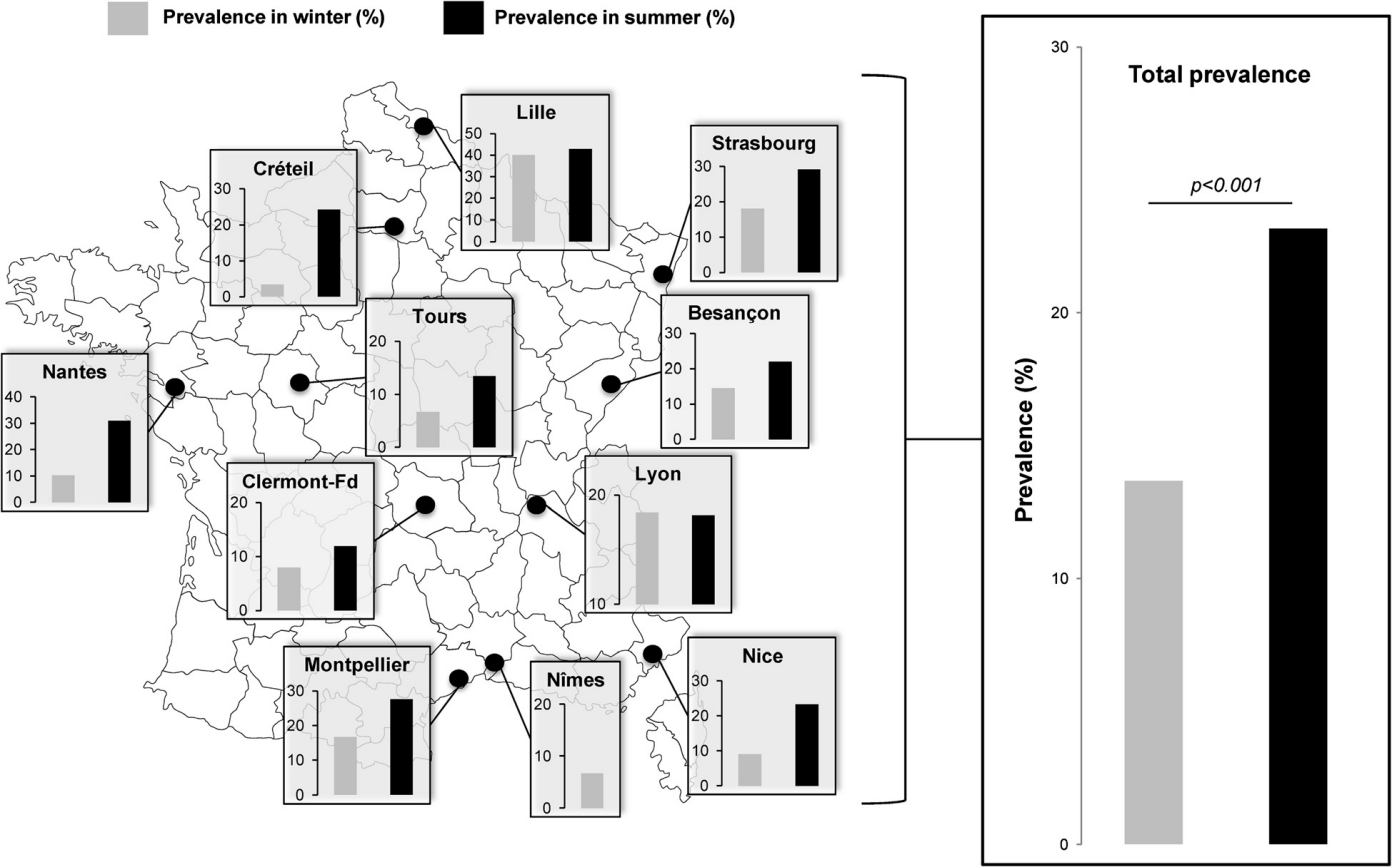
Location of the 11 French centers and seasonal prevalence of *Blastocystis* sp. by center. Prevalence data were obtained from the 788 enrolled patients. The map of France was obtained from the website Servier Medical Art (http://www.servier.fr/smart/banque-dimages-powerpoint)

This cross-sectional study was conducted in France between December 2012 and September 2013 and involved the parasitology-mycology medical laboratories of 11 teaching hospitals (Besançon, Clermont-Ferrand, Créteil, Lille, Lyon, Montpellier, Nantes, Nice, Nîmes, Strasbourg and Tours) throughout France (Fig. 1). As part of the study, each laboratory randomly selected 21 to 50 stool samples in both winter (from December 2012 to February 2013) and summer (from July 2013 to September 2013) to subsequently evaluate potential seasonal variations in the prevalence (percentage of subjects infected) of the parasite. All these samples (1 sample per patient) were collected at each participating center during routine clinical procedures. A total of 788 subjects followed up for different pathologies, with/without gastrointestinal symptoms were enrolled in this study. A standardized questionnaire was designed to collect information about each participating subject (sex, age, profession, recent travels and destinations, exposure to pets) as well as clinical data especially regarding immune status, presence of digestive symptoms (diarrhea, vomiting, bloating, constipation, and abdominal pain), and diagnosis of IBS or IBD (Inflammatory Bowel Disease). In addition, the observation of intestinal protozoan parasites (*Blastocystis* sp., amoebas, trichomonads, diplomonads, apicomplexa), fungi (microsporidia), and helminths by direct-light microscopy of fecal smears was also recorded, as well as digestive diseases of bacterial origin. For each subject, about 500 mg of fresh stools was collected and then homogenized by shaking in 1.5 ml of Stool Transport and Recovery (S.T.A.R.) buffer (Roche Diagnostics, Indianapolis, IN) (ratio 1:3 according to the manufacturer’s recommendations). Samples were stored and then transported at −20 °C to Lille for molecular screening and subtyping of *Blastocystis* sp.

### DNA extraction

Stool samples stored in the S.T.A.R. buffer were stirred and then centrifuged for 1 min at 1,000 xg. Total genomic DNA was extracted from 200 μL of the supernatant using the QiaAMP DNA Stool Mini Kit (Qiagen, Hilden, Germany) according to the manufacturer’s recommended procedures. The DNA was eluted in 100 μL of elution buffer (Qiagen) and stored at −20 °C until use.

### Detection and molecular subtyping of *Blastocystis* sp

For each sample, 2 μL of extracted DNA was subjected to real-time quantitative PCR (qPCR) assay to detect and subtype *Blastocystis* sp. qPCR was carried out using the *Blastocystis*-specific primer pair BL18SPPF1 (5’-AGTAGTCATACGCTCGTCTCAAA-3’)/BL18SR2PP (5’-TCTTCGTTACCCGTTACTGC-3’) targeting the SSU rRNA coding region as previously described [9]. DNA extraction controls (isolation of DNAs without stool and from a *Blastocystis* sp.-negative stool) subsequently used in qPCR assays and positive (DNA obtained from *Blastocystis* sp. ST4 or ST7 cultures) and negative (DNA matrix replaced by water) qPCR controls were performed. qPCR product from each positive sample was purified and sequenced in both strands by Genoscreen (Lille, France) or Beckman Coulter Genomics (Essex, United Kingdom). The SSU rRNA gene sequences obtained in this study were deposited in GenBank under accession numbers KU158944 to KU159084 (see Additional file 1). Obtained sequences were compared with all *Blastocystis* sp. homologous sequences available from the National Centre for Biotechnology Information (NCBI) using the Basic Local Alignment Search Tool (BLAST) program. STs were identified by determining the exact match or closest similarity against all known *Blastocystis* sp. STs. For two samples, sequence chromatograms analysis revealed the presence of double traces, suggesting mixed infection by different STs.

### Statistical analysis

All analyses were performed using the Stata statistical software (version 13, StataCorp, College Station, US). The categorical data were expressed as the number of patients and associated percentages, whereas the quantitative data were expressed as the mean and associated standard deviation according to the statistical distribution (assumption of normality studied using Shapiro-Wilk’s test). Comparisons between independent groups were performed by Chi-square or Fisher’s exact tests for categorical parameters and by Student’s t or Mann-Whitney tests when *t*-test conditions were not respected (normality and homoscedasticity studied by the Fisher-Snedecor test) for quantitative variables. The multivariate analysis was performed by stepwise approach according to univariate results and clinical relevance. A random-effects logistic regression was applied to determine parameters associated with the prevalence of *Blastocystis* sp., taking into account between and within center variability. Results were reported as odds-ratio (noted OR) and 95 % confidence intervals. P-values of 0.05 or below were considered significant (two-sided). Due to multiple comparisons, the type-I-error correction was considered when appropriate. So, the Marascuillo’s procedure was performed after Chi-square test (for example prevalence of *Blastocystis* sp. among age groups).

## Results

### Analysis of the cohort

Stool samples were collected from a total of 788 patients (Table 1). Among them, 417 were recruited during winter and 371 in summer. The sex ratio (M/F) was 1.37 and the age of the participants (7 missing data) was between 7 months and 95 years (mean age of 45.7 ± 21.3 years). Epidemiological records revealed that 178 patients had a recent history of travel outside France (i.e. during the last 12 months). Regarding the immune statuses of the patients, 351 were immunocompetent and 378 were immunocompromised (59 missing data). Among immunocompromised patients, 58 were positive for Human Immunodeficiency Virus (HIV) infection, 95 received a solid organ transplant, 131 received immunosuppressive therapy, 65 received a bone marrow transplant, and 34 suffered from tumorigenic processes. Forty patients suffered from chronic intestinal disease with 25 subjects presenting IBD and 15 IBS. Among this cohort, 502 patients presented digestive symptoms, such as diarrhea (52.4 %), abdominal pain (29.9 %), bloating (6.7 %), constipation (3.6 %) and vomiting (3.3 %), and 234 subjects were asymptomatic (52 missing data).

**Table 1:** Prevalence and ST distribution of *Blastocystis* sp. by center and among the total cohort

Center^a^	Samples (*n*)	Positive samples (*n*)	Prevalence (%)	*Blastocystis* sp. STs						Mixed infection (*n*)
				ST1	ST2	ST3	ST4	ST6	ST7	
Besançon (W)	48	7	14.6	-	2	3	2	-	-	-
Besançon (S)	41	9	22.0	1	1	2	4	-	-	1
Besançon (T)	89	16	18.0	1	3	5	6	-	-	1
Clermont-Ferrand (W)	50	4	8.0	1	-	1	1	1	-	-
Clermont-Ferrand (S)	50	6	12.0	-	-	2	4	-	-	-
Clermont-Ferrand (T)	100	10	10.0	1	-	3	5	1	-	-
Créteil (W)	29	1	3.4	1	-	-	-	-	-	-
Créteil (S)	29	7	24.1	1	-	6	-	-	-	-
Créteil (T)	58	8	13.8	2	-	6	-	-	-	-
Lille (W)	30	12	40.0	4	1	3	3	1	-	-
Lille (S)	21	9	42.9	2	1	3	3	-	-	-
Lille (T)	51	21	41.2	6	2	6	6	1	-	-
Lyon (W)	38	7	18.4	3	1	2	-	-	1	-
Lyon (S)	44	8	18.2	1	2	4	-	1	-	-
Lyon (T)	82	15	18.3	4	3	6	-	1	1	-
Montpellier (W)	30	5	16.7	-	2	3	-	-	-	-
Montpellier (S)	29	8	27.6	1	2	3	2	-	-	-
Montpellier (T)	59	13	22.0	1	4	6	2	-	-	-
Nantes (W)	49	5	10.2	-	-	3	1	-	1	-
Nantes (S)	42	13	30.1	3	1	3	4	-	1	1
Nantes (T)	91	18	19.8	3	1	6	5	-	2	1
Nice (W)	33	3	9.1	-	1	2	-	-	-	-
Nice (S)	30	7	23.3	-	1	6	-	-	-	-
Nice (T)	63	10	15.9	-	2	8	-	-	-	-
Nîmes (W)	30	2	6.7	-	-	2	-	-	-	-
Nîmes (S)	-	-	-	-	-	-	-	-	-	-
Nîmes (T)	30	2	6.7	-	-	2	-	-	-	-
Strasbourg (W)	50	9	18.0	3	-	4	2	-	-	-
Strasbourg (S)	48	14	29.2	3	3	7	1	-	-	-
Strasbourg (T)	98	23	23.5	6	3	11	3	-	-	-
Tours (W)	30	2	6.7	2	-	-	-	-	-	-
Tours (S)	37	5	13.5	2	-	2	1	-	-	-
Tours (T)	67	7	10.4	4	-	2	1	-	-	-
Total (W)	417	57	13.7	14	7	23	9	2	2	0
Total (S)	371	86	23.2	14	11	38	19	1	1	2
Grand total	788	143	18.1	28	18	61	28	3	3	2

^a^ *W* winter, *S* summer, *T* total

### Screening for *Blastocystis* sp. using qPCR

**Fig. 2:**
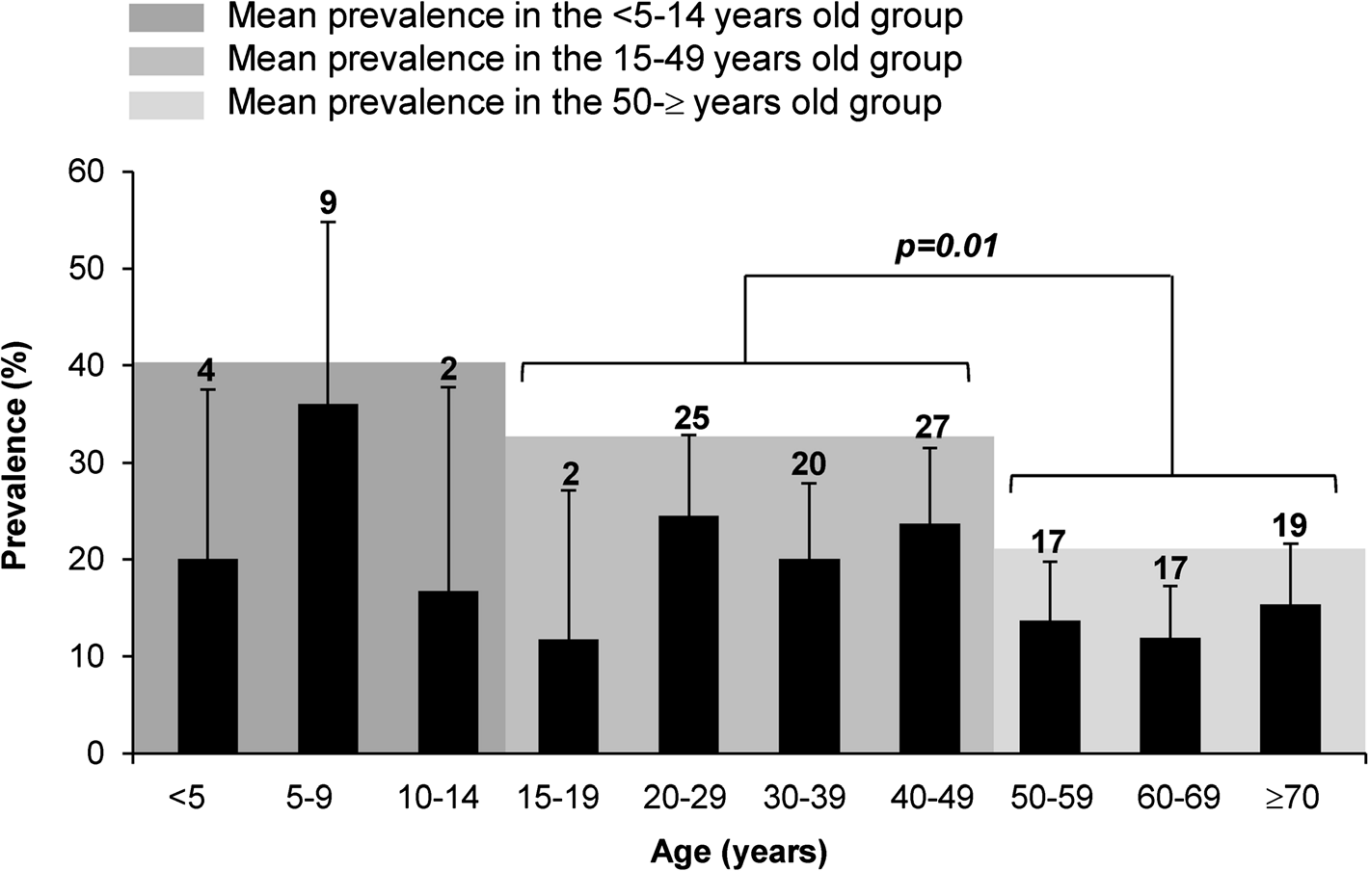
Age distribution of patients with blastocystosis (*n* = 142)

The overall prevalence of *Blastocystis* sp. was shown to be 18.1 % (143/788) in our study using qPCR (Table 1). The sensitivity of direct-light microscopy of fecal smears was only 45.8 % compared to the molecular method. All samples positive by microscopy were positive by the PCR assay. The overall prevalence determined by qPCR ranged from 10.0 % (Clermont-Ferrand) to 41.2 % (Lille) (Fig. 1 and Table 1). The overall prevalence in summer was significantly higher than in winter (23.2 % versus 13.7 %, *p < 0.001*) (Fig. 1 and Table 1). The difference in prevalence between males (19.5 %) and females (16.6 %) was not significant (*p = 0.29*). The mean age of *Blastocystis* sp.-infected patients was significantly lower when compared to the age in *Blastocystis* sp.-free subjects (43.0 ± 21.1 years versus 48.9 ± 21.1 years; Student’s *t*-test, *p = 0.003*). Subgroup analysis (Fig. 2) revealed that the prevalence of *Blastocystis* sp. was 26.3 % among subjects aged 0–14 years (15/57), 22.2 % in the group aged 15–49 years (74/333), and 13.6 % in patients aged over 50 years (53/391). However, while the prevalence of *Blastocystis* sp. in children was higher, with a peak between 5 and 9 years (36 %, Fig. 2), the statistical analysis only confirmed that subjects aged 15 to 49 years showed a higher prevalence than subjects aged over 50 years (*p = 0.01*, Marascuillo procedure; see also Additional file 2).

Among all the patients, 70 were found to be infected with other enteric parasites, either protozoans or helminths (see Additional file 3). The prevalence of *Blastocystis* sp. reached 40 % in those patients (28/70), compared to 16.2 % in subjects not infected with other enteric parasites (*p < 0.001*). Two additional patients presented bacterial infections with *Clostridium* and *Salmonella*, respectively.

Univariate and multivariate analyses highlighted that patients traveling during the last 12 months had a higher prevalence of *Blastocystis* sp. than non-travellers (27.5 versus 14.7 %, univariate: *p < 0.001*, multivariate logistic model: OR = 1.90 [1.18; 3.05], *p = 0.009*). A history of two or more travels during the previous year was associated with a higher prevalence, reaching 33.3 % (*p = 0.43*). Even though the travel destination was recorded (Africa, South America, Asia, North America, Oceania, and Europe), no significant difference between destinations and the prevalence of *Blastocystis* sp. was identified.

The socioeconomic level of all subjects was also recorded, and different professional classes were distinguished, including students, workers, executives, the unemployed and pensioners. The prevalence of *Blastocystis* sp. was not significantly different between the different professional classes. Food handlers and pet owners did not show a significantly higher prevalence than other individuals (22.2 versus 18.8 %, *p = 0.57* and 21.9 versus 19.7 %, *p = 0.61*, respectively). The prevalence of *Blastocystis* sp. in immunocompromised subjects was significantly lower than in immunocompetent patients (12.4 versus 24.2 %, *p < 0.001*). Within immunocompromised subjects, patients presenting with HIV, solid organ transplants, immunosuppressive therapy, solid cancer, and bone marrow transplants were distinguished. Subgroup analyses confirmed that the prevalence of *Blastocystis* sp. was significantly lower in patients receiving immunosuppressive treatment (8.4 %, *p = 0.001*) and with bone marrow transplant (7.7 %, *p = 0.02*) but not significantly lower in the other subgroups. The prevalence of *Blastocystis* sp. in patients suffering from IBD and IBS was 12 and 20 %, respectively, compared to 18.4 % in patients without a history of chronic bowel diseases (*p = 0.41* and *p = 0.88*, respectively). Moreover, all IBS and IBD patients infected with *Blastocystis* sp. were not infected with other enteric parasites.

### *Blastocystis* sp. infection and digestive symptoms

**Fig. 3:**
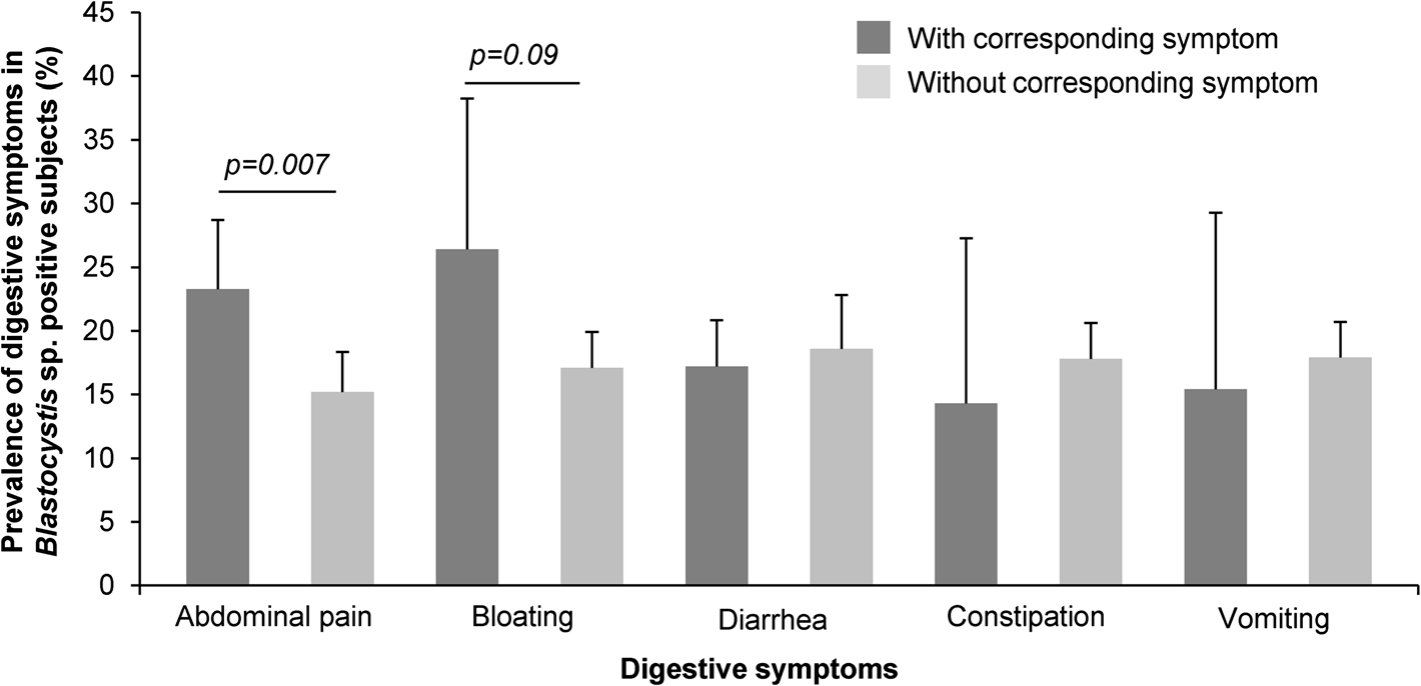
Distribution of digestive symptoms in symptomatic patients infected or not infected with *Blastocystis* sp. (*n* = 502)

The prevalence of *Blastocystis* sp. was not shown to be significantly higher in symptomatic patients (18.5 %, 93/502) than in asymptomatic carriers (16.2 %, 38/234). The digestive symptoms selected for the study were abdominal pain, bloating, diarrhea, constipation, and vomiting (Fig. 3). Within subjects positive for the parasite and presenting digestive symptoms, *Blastocystis* sp. infection was significantly associated with abdominal pain (23.3 % in patients with abdominal pain versus 15.7 % in patients without abdominal pain, *p = 0.007*). However, the most frequent symptom observed in symptomatic carriers was bloating, even if its prevalence in *Blastocystis* sp.-infected patients compared to non-carriers was not significant (26.4 versus 17.1 %, *p = 0.09*).

### Distribution of *Blastocystis* sp. STs

Among the 143 positive samples, 6 different STs were identified (ST1 to ST4, ST6 and ST7) (Table 1). Two patients had mixed infection (2/143, 1.4 %). Among the 141 patients with a single ST, ST3 was predominant (*n* = 61, 43.3 %), followed by ST1 and ST4 (both *n* = 28, 20.0 %), ST2 (*n* = 18, 12.8 %), ST6 and ST7 (both *n* = 3, 2.1 %) (Tables 1 and 2). The distribution of STs varied widely between the medical centers (Table 1), and no significant difference in the distribution of STs was found according to the season. Moreover, the distribution of STs between the symptomatic and asymptomatic groups of patients was almost similar. The genetic diversity among isolates belonging to the same ST was very low since 130/141 isolates (92.2 %) belonging to the different STs identified in the present study showed 99 to 100 % identity with homologous sequences available in databases for the same STs. Only 11 isolates belonging either to the ST1 or ST3 exhibited 96 to 98 % identity with homologous sequences. Therefore, numerous sequences of isolates belonging to the same ST were similar or closely related to each other.

**Table 2:** Total and seasonal distribution of *Blastocystis* sp. STs (*n* = 141)

*Blastocystis* sp. STs	ST1	ST2	ST3	ST4	ST6	ST7
Winter *n* (%)	14 (24.1)	7 (12.1)	23 (39.7)	9 (15.5)	2 (3.4)	2 (3.4)
Summer *n* (%)	14 (16.7)	11 (13.1)	38 (45.2)	19 (22.6)	1 (1.2)	1 (1.2)
Total *n* (%)	28 (20.0)	18 (12.8)	61 (43.3)	28 (20.0)	3 (2.1)	3 (2.1)

## Discussion

Little data are available in the literature regarding the prevalence of *Blastocystis* sp. in France and more generally in European countries. In France, the first two studies reported a prevalence of 3 and 6.1 % in two cohorts of 2,581 and 9,700 patients, respectively, by direct-light microscopy of fecal smears [19, 20]. More recently, the prevalence of *Blastocystis* sp. reached 14.5 % in a single-center study including 186 patients using a molecular assay [9]. Interestingly, this latter value is roughly similar to the prevalence of 18.1 % reported in the current samples using the same qPCR assay. By comparison, the prevalence reported in a few other neighbouring countries was 24.2 % in the Netherlands (*n* = 442) [5], 23 % in Denmark (*n* = 93) [22], 7.1 % in Italy (*n* = 5,351) [23], 7 % in Spain (*n* = 8,313) [24], and 6.9 and 3.9 % in the United Kingdom (*n* = 1,390 and *n* = 1,000, respectively) [25, 26] (Table 3). However, a comparison of the prevalence obtained from these various European studies remains generally uninformative due to the differences in the composition of the cohorts of patients and especially in the diagnostic tools. Indeed, apart from the Dutch and Danish studies, which were conducted using molecular tools and showed a prevalence similar to that of our survey, all other European epidemiological studies were performed using direct-light microscopy or in vitro culture, both methods being shown to be less sensitive than PCR [9]. The present study confirms this observation, since direct-light microscopy showed only 45.8 % sensitivity compared to the qPCR assay.

**Table 3:** Prevalence of *Blastocystis* sp. in European countries

Country	Region/city	Total number of patients	Method of detection	Prevalence	Reference
France	Grenoble	2,581	Direct-light microscopy	3.0 %	[19]
France	Paris	9,700	Direct-light microscopy	6.1 %	[20]
France	Clermont-Ferrand	186	qPCR	14.5 %	[9]
France	Multi-center study	788	qPCR	18.1 %	Present study
The Netherlands	Amsterdam	442	PCR	24.2 %	[5]
Denmark	Copenhagen	93	PCR	23.0 %	[22]
Italy	Rome	5,351	Direct-light microscopy	7,1 %	[23]
Spain	Catalonia	8,313	Direct-light microscopy	7.0 %	[24]
United Kingdom (Wales)	Aberystwyth	1,390	Direct-light microscopy	6.9 %	[25]
United Kingdom (Scotland)	Glasgow	1,000	In vitro culture and direct-light microscopy	3.9 %	[26]

Within the French centers, the prevalence of *Blastocystis* sp. ranged from 6.7 to 41.2 %. This variation may be naturally explained by differences in the composition of the respective cohorts from each center, but may also reflect differences in food habits and sources of drinking water in various geographic areas, which are also more or less rural and have different climate conditions. In future studies, the analysis of food and environmental samples in these regions, and especially the control of water sources regarding the presence of *Blastocystis* sp., might help identifying potential primary reservoirs of transmission. A key finding of our study was the seasonal impact on the prevalence of *Blastocystis* sp., which reached 23.2 % in summer compared to 13.7 % in winter. Interestingly, this seasonal pattern, already described in previous epidemiological surveys [26–29], was observed in 9 of the 10 French centers providing samples during both winter and summer. In France, this difference may be explained by changes in food habits according to the seasons, with an increased consumption of vegetables and fruits, drinks with ice cubes and ice creams in summer. Common water-based recreational activities may also be involved, since human fecal contamination was clearly shown to be correlated with *Blastocystis* sp. load in recreational rivers, suggesting a greater risk of infection by the parasite in summer [28, 29]. Moreover, frequent trips during the summer holidays and stays for instance in densely populated holiday centers could represent other risk factors for infection.

Among our overall population, gender was not identified as a potential risk factor associated with *Blastocystis* sp. infection, since the finding of a slightly higher prevalence of the parasite in males (19.5 %) than in females (16.6 %) was not statistically significant. By contrast, *Blastocystis* sp. showed a different age-related epidemiological pattern. The mean age was thus significantly lower in *Blastocystis* sp.-infected patients (43.0 ± 21.1 years) than in non-carriers of the infection (48.9 ± 21.1 years). In addition, the prevalence of *Blastocystis* sp. was significantly higher in subjects aged 15 to 49 years compared to those aged over 50 years (22.2 versus 16.6 %). On the other side, the prevalence of *Blastocystis* sp. was also not significantly higher in the age group 0 to 14 years (26.3 %) compared to older age classes, likely due to the too small number of children included in our study. Interestingly, an infection peak was shown between 5and 9 years of age, suggesting that children in this age category might be more at risk for *Blastocystis* sp. infection. In this regard, previous studies reported peaking prevalence of the parasite among groups aged under 10 years [27, 30–32]. Such a high rate may be due to inadequate toilet training and hygiene practices of school-children and cross-transmission through close personal contact. In a recent survey conducted in the Netherlands [5], a significantly higher prevalence of *Blastocystis* sp. was reported among patients with a history of recent travel, suggesting that trips to tropical and low-income countries may increase the risk of parasite infection. A similar conclusion was drawn from our study, since travel during the last 12 months was significantly associated with a higher prevalence of *Blastocystis* sp. (27.5 versus 14.7 % for non-travellers). Interestingly, the prevalence of the parasite reached 33.3 % in patients reporting at least two travels during the last year in countries at risk. Travellers should therefore follow food and water hygiene recommendations to prevent infection by *Blastocystis* sp.

Intestinal parasitic infections are among the leading causes of morbidity and mortality in patients infected with HIV. Consequently, the *Blastocystis* sp. detection as a possible pathogenic agent among immunocompromised patients continues to be debated. In this regard, the prevalence of *Blastocystis* sp. was previously found to be significantly higher in immunocompromised HIV patients, most presenting with diarrhea, than in HIV-seronegative controls [33–35]. Strikingly, *Blastocystis* sp. was the most commonly occurring parasite among the protozoans searched for in HIV-infected individuals [33, 34, 36, 37], with a prevalence reaching about 70 % in Jakarta, Indonesia [36]. In addition, a statistically significant association was shown between infection with *Blastocystis* sp. and the presence of digestive disorders among severely immunocompromised HIV-positive patients (with CD4+ T-cell counts < 200/μL) [33]. The prevalence of the parasite was also shown to be negatively correlated with the CD4+ cell count [36] and was significantly higher in HIV patients without antiretroviral therapy than among HIV-positive patients with treatment [38]. In immunocompromised patients presenting haematological malignancies, *Blastocystis* sp. was more frequently associated with gastrointestinal symptoms than in non-immunocompromised symptomatic patients [39]. This contrasted with a more recent French survey showing no correlation between digestive symptoms and immune status in patients presenting similar pathology [9]. All-in-all, immunodepression seems to be a factor of primary importance in the infection and pathogenic role of *Blastocystis* sp. However, in the present study, the prevalence of the parasite in immunocompromised subjects was significantly lower than in immunocompetent individuals (12.4 versus 24.2 %, respectively), especially in subgroups of patients receiving immunosuppressive therapy (8.4 %) or with bone marrow transplants (7.7 %). In our opinion, the controlled food diet recommended to these patients for the prevention of potential opportunistic infections along with antibiotic therapy such as metronidazole might have a negative impact on the prevalence of *Blastocystis* sp. In addition, a history of travel was shown above to be positively correlated with the prevalence of *Blastocystis* sp.. In fact, only 21.5 % of immunocompromised patients reported having travelled during the last 12 months, compared to 35.4 % of immunocompetent individuals.

To clarify the clinical relevance of *Blastocystis* sp, numerous studies were published related to the comparison of parasite prevalence between symptomatic and asymptomatic individuals [1–3]. If accumulating epidemiological studies suggested that *Blastocystis* sp. was associated with gastrointestinal disorders, numerous reports did not support this association. From our overall population, the prevalence of *Blastocystis* sp. was not significantly different between symptomatic and asymptomatic patients, what does not however prejudge the pathogenicity of various isolates. Within the symptomatic group, abdominal pain was reported significantly more frequently in *Blastocystis* sp. carriers, in agreement with earlier studies recording abdominal pain as one of the most common symptoms of blastocystosis [1]. Bloating was also attributed to blastocystosis in various studies [1]. This symptom was most frequently identified in *Blastocystis* sp.-positive subjects of our cohort, but was not significantly associated with parasite infection.

The prevalence of the parasite in patients suffering from chronic bowel disorders was also investigated, since recent studies suggested an association between *Blastocystis* sp. and IBS [2, 16], a functional bowel disorder with a prevalence ranging from 5 to 24 % in industrialized countries [18]. Based on a systematic review of the literature and a meta-analysis including previous epidemiological studies in IBS cohorts, it was shown that IBS patients had a relative risk of 2.34 of being infected with *Blastocystis* sp. when compared to non-IBS subjects [17]. However, in our study, the prevalence of the parasite was not significantly higher in IBS patients (20 %) compared to non-IBS subjects (18.5 %). Since the diagnosis of IBS is still difficult and requires a specific visit to a gastroenterologist, some patients in our study may not have been diagnosed as positive for IBS, which could impact our results. In parallel, according to various studies, the prevalence of *Blastocystis* sp. in IBD patients was reported to be lower or higher than in non-IBD subjects, which probably depends on the type of IBD (mainly Crohn’s disease or ulcerative colitis) [40, 41]. In our study, the parasite was identified less frequently in IBD subgroups (12 %), but not significantly less than in non-IBD patients (18.4 %). Unfortunately, subgroups analyses from different types of IBD were not performed because of the small size of the IBD cohort.

As part of our study, a total of 141 *Blastocystis* sp. isolates were subtyped to evaluate the ST distribution within our French cohort. ST3 was the most common ST (43.3 %), followed by ST1 and ST4 (20 %), ST2 (12.8 %), ST6 and ST7 (2.1 %). This distribution is nearly similar to that observed in a majority of geographical areas all over the world, including European countries [5, 22, 42], with a predominance of ST3, followed by ST1, ST2 or ST4, highlighting large-scale inter-human transmission [2, 4]. The identification of a few isolates in Lille, Lyon, Nantes, or Clermont-Ferrand belonging to ST6 or ST7 is most likely the result of zoonotic transmission, since both STs are considered avian STs [1–4, 10]. The distribution of some STs in the overall human population showed significant geographical variations, especially in relation to the ST4 [2, 4]. Our data thus confirmed that ST4 is commonly found in Europe [5, 22, 42] and especially in France [9], and is much less frequently detected or absent in Africa, America and Asia. This observation might perhaps be explained by the recently proposed emergence of ST4 in the human population in Europe [2]. The ST distribution was also variable between different French centers, although ST3 was predominant in 8 of the 11 centers. In two previous studies conducted in France, ST3 was the most frequent ST in Lille [21], whereas ST4 showed a higher prevalence in Clermont-Ferrand [9], in agreement with our survey. These patterns in ST distribution, as well as in prevalence of the parasite between centers, suggest that reservoirs and/or sources of contamination may differ from any geographical area of France to another. Until now, and for different reasons, the epidemiological data remain contradictory regarding the correlation between ST and the pathogenesis of *Blastocystis* sp. [1, 21]. In this regard, our study failed to provide any evidence for ST association with specific symptoms status.

## Conclusions

Our survey provides new insights into the epidemiology of *Blastocystis* sp. in industrialized countries through the first multi-center study conducted in France. Such a multi-center survey gives a more comprehensive view of the parasite situation in France, by obtaining data regarding the prevalence and ST distribution of the parasite in different geographical areas. From our overall data, age and a history of recent travel represent the principal reliable risk factors for acquiring this infection. A seasonal impact on the prevalence of *Blastocystis* sp. is also highlighted, with a higher prevalence in summer. Large variations observed in the prevalence and ST distribution of the parasite between French regions suggest various reservoirs and sources of transmission. Further studies in other European countries, with multi-center recruitment of patients, will clearly be required to establish a complete mapping of the prevalence and ST distribution of *Blastocystis* sp. in this continent and to improve our understanding of the circulation of the parasite within the European population.

## Additional files

Additional file 1: Isolation source (center), season of collect, ST identification and GenBank accession number of *Blastocystis* sp. isolates characterized in our study. (DOCX 20 kb) Additional file 2: Post hoc comparison of the prevalence of *Blastocystis* sp. between age groups. (DOCX 14 kb) Additional file 3: Presence of other enteric parasites (protozoans and helminths) and digestive symptoms in the 143 French patients infected with *Blastocystis* sp. (DOCX 23 kb)

## Abbreviations

HIV: Human immunodeficiency virus
IBD: Inflammatory bowel disease
IBS: Irritable bowel syndrome
OR: Odds-ratio
qPCR: Real-time quantitative PCR
ST: Subtype
