# Supplementary material for: Prevalence, risk factors for infection and subtype distribution of the intestinal parasite Blastocystis sp. from a large-scale multi-center study in France
Source: BMC Infect Dis. 2016 Aug 26;16(1):451. doi: 10.1186/s12879-016-1776-8 (PMC5002209; doi:10.1186/s12879-016-1776-8)
Supplement: Additional file 1: — Isolation source (center), season of collect, ST identification and GenBank accession number of Blastocystis sp. isolates characterized in our study. (DOCX 20 kb) [file 12879_2016_1776_MOESM1_ESM.docx]

**Additional file 1. Isolation source (center), season of collect, ST identification and GenBank accession number of *Blastocystis* sp. isolates characterized in our study.**

| Isolate | Center | Season^a^ | *Blastocystis* sp. ST | GenBank accession number |
| --- | --- | --- | --- | --- |
| HT1 | Tours | W | ST1 | KU158944 |
| HT5 | Tours | W | ST1 | KU158945 |
| ET4 | Tours | S | ST3 | KU158946 |
| ET12 | Tours | S | ST1 | KU158947 |
| ET27 | Tours | S | ST3 | KU158948 |
| ET35 | Tours | S | ST4 | KU158949 |
| ET40 | Tours | S | ST1 | KU158950 |
| HBS14 | Besançon | W | ST4 | KU158951 |
| HBS22 | Besançon | W | ST2 | KU158952 |
| HBS26 | Besançon | W | ST2 | KU158953 |
| HBS27 | Besançon | W | ST3 | KU158954 |
| HBS30 | Besançon | W | ST4 | KU158955 |
| HBS41 | Besançon | W | ST3 | KU158956 |
| HBS43 | Besançon | W | ST3 | KU158957 |
| EBS5 | Besançon | S | ST3 | KU158958 |
| EBS7 | Besançon | S | ST2 | KU158959 |
| EBS15 | Besançon | S | ST4 | KU158960 |
| EBS19 | Besançon | S | ST3 | KU158961 |
| EBS27 | Besançon | S | ST4 | KU158962 |
| EBS30 | Besançon | S | ST4 | KU158963 |
| EBS33 | Besançon | S | ST4 | KU158964 |
| EBS42 | Besançon | S | ST1 | KU158965 |
| HLi1 | Lille | W | ST3 | KU158966 |
| HLi2 | Lille | W | ST1 | KU158967 |
| HLi8 | Lille | W | ST4 | KU158968 |
| HLi9 | Lille | W | ST1 | KU158969 |
| HLi13 | Lille | W | ST6 | KU158970 |
| HLi14 | Lille | W | ST4 | KU158971 |
| HLi15 | Lille | W | ST2 | KU158972 |
| HLi18 | Lille | W | ST3 | KU158973 |
| HLi20 | Lille | W | ST3 | KU158974 |
| HLi25 | Lille | W | ST1 | KU158975 |
| HLi29 | Lille | W | ST1 | KU158976 |
| HLi30 | Lille | W | ST4 | KU158977 |
| ELi1 | Lille | S | ST3 | KU158978 |
| ELi2 | Lille | S | ST1 | KU158979 |
| ELi3 | Lille | S | ST4 | KU158980 |
| ELi4 | Lille | S | ST2 | KU158981 |
| ELi8 | Lille | S | ST1 | KU158982 |
| ELi10 | Lille | S | ST3 | KU158983 |
| ELi12 | Lille | S | ST4 | KU158984 |
| ELi17 | Lille | S | ST3 | KU158985 |
| ELi18 | Lille | S | ST4 | KU158986 |
| HMon11 | Montpellier | W | ST2 | KU158987 |
| HMon13 | Montpellier | W | ST3 | KU158988 |
| HMon15 | Montpellier | W | ST3 | KU158989 |
| HMon28 | Montpellier | W | ST2 | KU158990 |
| HMon29 | Montpellier | W | ST3 | KU158991 |
| EMon3 | Montpellier | S | ST1 | KU158992 |
| EMon4 | Montpellier | S | ST3 | KU158993 |
| EMon5 | Montpellier | S | ST3 | KU158994 |
| EMon8 | Montpellier | S | ST4 | KU158995 |
| EMon17 | Montpellier | S | ST4 | KU158996 |
| EMon18 | Montpellier | S | ST3 | KU158997 |
| EMon25 | Montpellier | S | ST2 | KU158998 |
| EMon26 | Montpellier | S | ST2 | KU158999 |
| HNIM7 | Nîmes | W | ST3 | KU159000 |
| HNIM25 | Nîmes | W | ST3 | KU159001 |
| HNC13 | Nice | W | ST3 | KU159002 |
| HNC21 | Nice | W | ST3 | KU159003 |
| HNC36 | Nice | W | ST2 | KU159004 |
| ENC5 | Nice | S | ST3 | KU159005 |
| ENC8 | Nice | S | ST3 | KU159006 |
| ENC9 | Nice | S | ST3 | KU159007 |
| ENC11 | Nice | S | ST3 | KU159008 |
| ENC12 | Nice | S | ST3 | KU159009 |
| ENC13 | Nice | S | ST2 | KU159010 |
| ENC22 | Nice | S | ST3 | KU159011 |
| HSTB3 | Strasbourg | W | ST1 | KU159012 |
| HSTB4 | Strasbourg | W | ST1 | KU159013 |
| HSTB6 | Strasbourg | W | ST3 | KU159014 |
| HSTB12 | Strasbourg | W | ST4 | KU159015 |
| HSTB14 | Strasbourg | W | ST4 | KU159016 |
| HSTB18 | Strasbourg | W | ST1 | KU159017 |
| HSTB22 | Strasbourg | W | ST3 | KU159018 |
| HSTB28 | Strasbourg | W | ST3 | KU159019 |
| HSTB31 | Strasbourg | W | ST3 | KU159020 |
| ESTB17 | Strasbourg | S | ST3 | KU159021 |
| ESTB19 | Strasbourg | S | ST2 | KU159022 |
| ESTB20 | Strasbourg | S | ST2 | KU159023 |
| ESTB22 | Strasbourg | S | ST3 | KU159024 |
| ESTB29 | Strasbourg | S | ST2 | KU159025 |
| ESTB32 | Strasbourg | S | ST1 | KU159026 |
| ESTB34 | Strasbourg | S | ST4 | KU159027 |
| ESTB35 | Strasbourg | S | ST3 | KU159028 |
| ESTB38 | Strasbourg | S | ST1 | KU159029 |
| ESTB39 | Strasbourg | S | ST3 | KU159030 |
| ESTB40 | Strasbourg | S | ST3 | KU159031 |
| ESTB44 | Strasbourg | S | ST3 | KU159032 |
| ESTB49 | Strasbourg | S | ST1 | KU159033 |
| ESTB50 | Strasbourg | S | ST3 | KU159034 |
| HLY2 | Lyon | W | ST1 | KU159035 |
| HLY5 | Lyon | W | ST2 | KU159036 |
| HLY27 | Lyon | W | ST1 | KU159037 |
| HLY29 | Lyon | W | ST7 | KU159038 |
| HLY32 | Lyon | W | ST1 | KU159039 |
| HLY35 | Lyon | W | ST3 | KU159040 |
| HLY36 | Lyon | W | ST3 | KU159041 |
| ELY17 | Lyon | S | ST2 | KU159042 |
| ELY23 | Lyon | S | ST3 | KU159043 |
| ELY28 | Lyon | S | ST1 | KU159044 |
| ELY34 | Lyon | S | ST2 | KU159045 |
| ELY35 | Lyon | S | ST3 | KU159046 |
| ELY36 | Lyon | S | ST3 | KU159047 |
| ELY45 | Lyon | S | ST3 | KU159048 |
| ELY46 | Lyon | S | ST6 | KU159049 |
| HNA4 | Nantes | W | ST7 | KU159050 |
| HNA12 | Nantes | W | ST3 | KU159051 |
| HNA32 | Nantes | W | ST3 | KU159052 |
| HNA36 | Nantes | W | ST3 | KU159053 |
| HNA38 | Nantes | W | ST4 | KU159054 |
| ENA5 | Nantes | S | ST3 | KU159055 |
| ENA10 | Nantes | S | ST1 | KU159056 |
| ENA17 | Nantes | S | ST3 | KU159057 |
| ENA20 | Nantes | S | ST4 | KU159058 |
| ENA25 | Nantes | S | ST1 | KU159059 |
| ENA27 | Nantes | S | ST1 | KU159060 |
| ENA33 | Nantes | S | ST7 | KU159061 |
| ENA34 | Nantes | S | ST3 | KU159062 |
| ENA36 | Nantes | S | ST4 | KU159063 |
| ENA37 | Nantes | S | ST4 | KU159064 |
| ENA39 | Nantes | S | ST2 | KU159065 |
| ENA41 | Nantes | S | ST4 | KU159066 |
| HCF11 | Clermont-Ferrand | W | ST6 | KU159067 |
| HCF19 | Clermont-Ferrand | W | ST1 | KU159068 |
| HCF30 | Clermont-Ferrand | W | ST4 | KU159069 |
| HCF50 | Clermont-Ferrand | W | ST3 | KU159070 |
| ECF52 | Clermont-Ferrand | S | ST4 | KU159071 |
| ECF54 | Clermont-Ferrand | S | ST3 | KU159072 |
| ECF64 | Clermont-Ferrand | S | ST4 | KU159073 |
| ECF71 | Clermont-Ferrand | S | ST4 | KU159074 |
| ECF72 | Clermont-Ferrand | S | ST4 | KU159075 |
| ECF85 | Clermont-Ferrand | S | ST3 | KU159076 |
| HCR1 | Créteil | W | ST1 | KU159077 |
| ECR32 | Créteil | S | ST3 | KU159078 |
| ECR34 | Créteil | S | ST3 | KU159079 |
| ECR35 | Créteil | S | ST3 | KU159080 |
| ECR38 | Créteil | S | ST3 | KU159081 |
| ECR42 | Créteil | S | ST3 | KU159082 |
| ECR45 | Créteil | S | ST3 | KU159083 |
| ECR50 | Créteil | S | ST1 | KU159084 |

^a^W, winter ; S, summer
