# Supplementary material for: Prevalence, risk factors for infection and subtype distribution of the intestinal parasite Blastocystis sp. from a large-scale multi-center study in France
Source: BMC Infect Dis. 2016 Aug 26;16(1):451. doi: 10.1186/s12879-016-1776-8 (PMC5002209; doi:10.1186/s12879-016-1776-8)
Supplement: Additional file 2: — Post hoc comparison of the prevalence of Blastocystis sp. between age groups. (DOCX 14 kb) [file 12879_2016_1776_MOESM2_ESM.docx]

**Additional file 2. Post hoc comparison of the prevalence of *Blastocystis* sp. between age groups.**

| *p-values* | <15 years (26.3%) | 15-49 years (22.2%) | ≥ 50 years (13.6%) |
| --- | --- | --- | --- |
| <15 years (26.3%) | NA^a^ | 0.78 | 0.11 |
| 15-49 years (22.2%) | 0.78 | NA | 0.01 |
| ≥ 50 years (13.6%) | 0.11 | 0.01 | NA |

^a^ NA, Not achieved
