# Supplementary material for: Prevalence, risk factors for infection and subtype distribution of the intestinal parasite Blastocystis sp. from a large-scale multi-center study in France
Source: BMC Infect Dis. 2016 Aug 26;16(1):451. doi: 10.1186/s12879-016-1776-8 (PMC5002209; doi:10.1186/s12879-016-1776-8)
Supplement: Additional file 3: — Presence of other enteric parasites (protozoans and helminths) and digestive symptoms in the 143 French patients infected with Blastocystis sp. (DOCX 23 kb) [file 12879_2016_1776_MOESM3_ESM.docx]

**Additional file 3. Presence of other enteric parasites (protozoans and helminths) and digestive symptoms in the 143 French patients infected with *Blastocystis* sp.**

| Patients | *Blastocystis* sp. ST | Other parasites identified by direct-light microscopy | Digestive symptoms^a^ |
| --- | --- | --- | --- |
| HT1 | ST1 | *Entamoeba coli, Endolimax nana* | - |
| HT5 | ST1 | - | - |
| HBS14 | ST4 | - | + |
| HBS22 | ST2 | - | + |
| HBS26 | ST2 | - | - |
| HBS27 | ST3 | - | - |
| HBS30 | ST4 | *Enterobius vermicularis* | + |
| HBS41 | ST3 | - | - |
| HBS43 | ST3 | *E. coli, E. nana* | + |
| HNA4 | ST7 | - | - |
| HNA12 | ST3 | *E. coli* | - |
| HNA32 | ST3 | *E. coli, Schistosoma mansoni* | - |
| HNA36 | ST3 | - | + |
| HNA38 | ST4 | - | + |
| HLi1 | ST3 | *E. histolytica, E. dispar* | + |
| HLi2 | ST1 | *G. intestinalis, E. histolytica / dispar, Trichuris trichiura* | + |
| HLi8 | ST4 | - | + |
| HLi9 | ST1 | - | - |
| HLi13 | ST6 | - | + |
| HLi14 | ST4 | *Strongyloïdes stercoralis, Ancylostoma* sp. | + |
| HLi15 | ST2 | *E. coli, E. histolityca, E. nana* | - |
| HLi18 | ST3 | - | - |
| HLi20 | ST3 | - | + |
| HLi25 | ST1 | - | + |
| HLi29 | ST1 | - | + |
| HLi30 | ST4 | - | + |
| HLY2 | ST1 | - | - |
| HLY5 | ST2 | - | + |
| HLY27 | ST1 | *E. histolytica, E. nana* | - |
| HLY29 | ST7 | - | + |
| HLY32 | ST1 | - | + |
| HLY35 | ST3 | - | + |
| HLY36 | ST3 | - | - |
| HMon11 | ST2 | - | + |
| HMon13 | ST3 | - | + |
| HMon15 | ST3 | - | - |
| HMon28 | ST2 | - | + |
| HMon29 | ST3 | *Hymenolepis nana, E.* *nana, E. coli* | + |
| HNC13 | ST3 | - | + |
| HNC21 | ST3 | *Schistosoma haematobium* | - |
| HNC36 | ST2 | *G. intestinalis* | + |
| HSTB3 | ST1 | - | - |
| HSTB4 | ST1 | *Dientamoeba fragilis* | + |
| HSTB6 | ST3 | - | - |
| HSTB12 | ST4 | - | + |
| HSTB14 | ST4 | - | + |
| HSTB18 | ST1 | *Cryptosporidium* sp., *E. dispar* | + |
| HSTB22 | ST3 | *D. fragilis* | + |
| HSTB28 | ST3 | - | - |
| HSTB31 | ST3 | - | - |
| HCF11 | ST6 | - | - |
| HCF19 | ST1 | - | + |
| HCF30 | ST4 | - | + |
| HCF50 | ST3 | - | + |
| HNIM7 | ST3 | - | + |
| HNIM25 | ST3 | - | + |
| HCR1 | ST1 | - | + |
| ET4 | ST3 | *G. intestinalis* | + |
| ET12 | ST1 | - | - |
| ET27 | ST3 | - | - |
| ET35 | ST4 | - | + |
| ET40 | ST1 | *Cryptosporidium* sp. | + |
| EBS5 | ST3 | - | + |
| EBS7 | ST2 | - | - |
| EBS15 | ST4 | - | - |
| EBS19 | ST3 | - | + |
| EBS26 | Mixed infection | - | + |
| EBS27 | ST4 | - | + |
| EBS30 | ST4 | - | + |
| EBS33 | ST4 | - | - |
| EBS42 | ST1 | - | - |
| ENA5 | ST3 | - | + |
| ENA10 | ST1 | *E. nana, E. coli* | + |
| ENA17 | ST3 | *E. coli* | + |
| ENA20 | ST4 | - | + |
| ENA25 | ST1 | *E. coli*, *E. dispar* or *E.* *histolytica*, *E. hartmanii*, *Pentatrichomonas hominis* | - |
| ENA27 | ST1 | - | - |
| ENA33 | ST7 | - | NA |
| ENA34 | ST3 | - | + |
| ENA36 | ST4 | - | - |
| ENA37 | ST4 | - | + |
| ENA39 | ST2 | - | + |
| ENA41 | ST4 | - | + |
| ENA42 | Mixed infection | - | NA |
| ELi1 | ST3 | - | + |
| ELi2 | ST1 | - | NA |
| ELi3 | ST4 | - | NA |
| ELi4 | ST2 | - | + |
| ELi8 | ST1 | - | + |
| ELi10 | ST3 | *E. nana, Pseudolimax butschlii* | + |
| ELi12 | ST4 | - | + |
| ELi17 | ST3 | - | NA |
| ELi18 | ST4 | - | + |
| ELY17 | ST2 | - | NA |
| ELY23 | ST3 | - | + |
| ELY28 | ST1 | - | + |
| ELY34 | ST2 | - | + |
| ELY35 | ST3 | - | + |
| ELY36 | ST3 | - | + |
| ELY45 | ST3 | - | + |
| ELY46 | ST6 | - | + |
| EMon3 | ST1 | *E. nana, E. coli* | + |
| EMon4 | ST3 | *Cyclospora cayetanensis* | + |
| EMon5 | ST3 | - | + |
| EMon8 | ST4 | - | NA |
| EMon17 | ST4 | - | + |
| EMon18 | ST3 | - | + |
| EMon25 | ST2 | - | NA |
| EMon26 | ST2 | - | NA |
| ENC5 | ST3 | - | + |
| ENC8 | ST3 | - | + |
| ENC9 | ST3 | - | + |
| ENC11 | ST3 | *Schistosoma* sp. | + |
| ENC12 | ST3 | - | + |
| ENC13 | ST2 | - | + |
| ENC22 | ST3 | - | + |
| ESTB17 | ST3 | - | - |
| ESTB19 | ST2 | - | - |
| ESTB20 | ST2 | - | NA |
| ESTB22 | ST3 | - | NA |
| ESTB29 | ST2 | - | + |
| ESTB32 | ST1 | - | + |
| ESTB34 | ST4 | - | NA |
| ESTB35 | ST3 | - | + |
| ESTB38 | ST1 | - | + |
| ESTB39 | ST3 | *Sarcocystis hominis* | + |
| ESTB40 | ST3 | - | + |
| ESTB44 | ST3 | - | NA |
| ESTB49 | ST1 | - | + |
| ESTB50 | ST3 | - | + |
| ECF2 | ST4 | - | + |
| ECF4 | ST3 | *G. intestinalis* | + |
| ECF14 | ST4 | - | - |
| ECF21 | ST4 | - | + |
| ECF22 | ST4 | - | + |
| ECF35 | ST3 | - | - |
| ECR32 | ST3 | - | + |
| ECR34 | ST3 | - | - |
| ECR35 | ST3 | - | - |
| ECR38 | ST3 | - | - |
| ECR42 | ST3 | - | - |
| ECR45 | ST3 | *Cryptosporidium* sp. | + |
| ECR50 | ST1 | - | + |

^a^ NA, not available
